# Radial Head Fractures: Is the Mason Classification Still Effective Today? A Large-Sample Validation of Intra- and Inter-Observer Reliability

**DOI:** 10.3390/jcm14207252

**Published:** 2025-10-14

**Authors:** Filippo Calderazzi, Davide Donelli, Alessandro Marinelli, Paolo Bastia, Cristina Galavotti, Alessandro Nosenzo, Enricomaria Lunini, Alessandra Maresca, Giorgio Concari, Corrado Ciatti

**Affiliations:** 1 Department of Medicine and Surgery, Orthopaedic Clinic, Maggiore Hospital, University of Parma, 43126 Parma, Italy; filippo.calderazzi@icloud.com; 2 Cardiology Unit, Department of Cardiothoracic and Vascular Diseases, Maggiore Hospital, University of Parma, 43126 Parma, Italy; donelli.davide@gmail.com; 3 Shoulder and Elbow Unit, IRCCS Rizzoli, 40100 Bologna, Italy; alessandro.marinelli@ior.it; 4 Department of Orthopaedic and Traumatology, Santa Chiara Hospital, 38122 Trento, Italy; pbastia88@gmail.com; 5 Department of Orthopaedic and Traumatology, ASST Cremona, 26100 Cremona, Italy; galavotticristina@gmail.com; 6 Department of Orthopaedic and Traumatology, Guastalla Civic Hospital, 42016 Guastalla, Italy; alessandronosenzo@libero.it; 7 Orthopaedic Department, ASST Metropolitan Hospital Niguarda, 20162 Milano, Italy; enricomaria.lunini@gmail.com; 8 Department of Orthopedics and Traumatology, Torrette Hospital, University of Marche, 60126 Ancona, Italy; alessandra.maresca@ospedaliriuniti.marche.it; 9 Department of Medicine and Surgery Operative Unit of Radiology, Maggiore Hospital, University of Parma, 43126 Parma, Italy; giorgio.concari@gmail.com; 10 Department of Orthopedics and Traumatology, Guglielmo da Saliceto Hospital, AUSL Piacenza, 29121 Piacenza, Italy

**Keywords:** radial head fractures, mason classification, multicenter study, inter-observer, intra-observer

## Abstract

**Introduction:** Various classifications of radial head fractures have been reported in the literature, most of them are based solely on conventional radiographic criteria. The Mason–Johnston classification, currently the most widely used system worldwide, is affected by the limitations of conventional radiographs. The aim of our study is to confirm or refute the low reliability and reproducibility of the Mason–Johnston classification. **Materials and Methods**: The study collected elbow X-rays showing radial head fractures from 2011 to 2021. Images were evaluated by eight orthopedic surgeons and one radiologist consultant from different hospitals for classification. The first phase assessed inter-observer agreement, comparing classifications among participants. After four months, the same images were randomly reordered and then reclassified to evaluate intra-observer agreement. A total of 90 elbow X-rays from 50 women and 40 men were analyzed. Inter- and intra-observer agreement was assessed using Fleiss’ kappa, Krippendorff alpha, and Cohen’s kappa. **Results**: Overall inter-observer agreement by unweighted Fleiss’ κ was moderate in both sessions (κ = 0.49 and κ = 0.50), with overall pairwise percent agreement 63% and prevalence- and bias-adjusted κ (PABAK, k = 4) ≈ 0.50. As an ordinal sensitivity analysis, Krippendorff’s α (ordinal) was 0.726 and 0.744, indicating substantial agreement. Type-specific reliability was moderate for Types II–III and higher for Type IV. Unweighted Cohen’s kappa coefficients were calculated to assess intra-observer agreement, demonstrating moderate to substantial levels of concordance. **Conclusions**: The Mason–Johnston classification shows moderate inter-observer reliability, especially for Types II–III, and moderate to substantial intra-observer agreement.

## 1. Introduction

Radial head fractures are among the most frequent injuries affecting the elbow joint, comprising nearly one-third of elbow fractures and around 2.5% of all adult skeletal injuries [1,2,3]. They occur commonly as a result of falls onto an outstretched hand or direct trauma and may be isolated or associated with complex ligamentous and bony injuries, including dislocations and coronoid fractures [4].

Accurate classification of these fractures is essential for guiding treatment decisions and prognostic expectations. The Mason classification, first introduced in 1954, has served as the foundational framework for categorizing radial head fractures into four types, with later modifications proposed by Johnston [5,6].

Despite its widespread clinical use, the Mason–Johnston classification has been criticized for its limited reproducibility and ability to inform treatment strategy [7,8,9,10]. In particular, its capacity to distinguish between subtle differences in fracture morphology is limited [11]. This is especially critical in Types II and III fractures, where treatment recommendations are often ambiguous [12].

This study aims to evaluate the inter- and intra-observer reliability of the Mason–Johnston classification using a large sample size and a multi-observer approach. The goal is to determine the utility of the Mason–Johnston system in the current diagnostic setting and explore the necessity for developing more comprehensive and imaging-informed classification systems.

## 2. Materials and Methods

This retrospective, observational, multicentric study was approved by the local ethics committee (study no. 789/2021/OSS*/Prot. 42502).

The first part of the study was conducted by the Study Manager; this phase regarded the selection, collection and observation of radiographic images extrapolated from the computer database of the diagnostic test visualization program used at our hospital (SuitEstensa; Esaote Biomedica, Indianapolis, IN, USA). All images analyzed concerned the elbow area and showed a fracture of the radial head, with or without associated bone lesions. Specifically, the first criterion for selecting the images from the computer program was based on the radiological diagnostic report associated with them (wording: “fracture of the radial head”). Only cases with complete radiographic documentation, including both true anteroposterior and true lateral views of the elbow, were considered for inclusion. Since the number of patients who underwent the Greenspan oblique view was too small, we decided not to include this projection in the inclusion criteria. The analyzed time frame was the period from 1 January 2011 to 30 April 2021. Images with poor resolution quality and from minor patients were excluded. The selected images were collected in special nominal folders for the exclusive use of the study manager. The names of the patients were then replaced with numerical codes, and the diagnostic images were submitted to the attention of the observers anonymously.

During the data extrapolation phase, the images of 90 patients were included in the study. They referred to 50 women and 40 men who reported a radial head fracture. The second part of the study was the image analysis and was conducted by eight orthopedic surgeons specialized in elbow trauma surgery and one musculoskeletal radiologist belonging to different centers. This part of the study was distinguished in two observation phases. First, the participants proceeded to classify the sample diagnostic images sent to them by the Study Manager. The patient names had been obscured and replaced with numeric codes. Each observer collected their results in a special spreadsheet which they then sent to the study manager for statistical analysis. The first observation phase was aimed at estimating the inter-observer agreement by comparing the data collected from each participant and evaluating the degree of agreement between the observers and therefore the reliability of the proposed classification.

After 4 months, the observers were called upon to re-classify the same radiographic images. However, the order of presentation of the X-ray images of each patient was changed randomly, generating a new sequence in order to minimize the risk of bias. Once the images were classified and the results were collected, a new statistical analysis was performed with the purpose to estimate the intra-observer agreement and therefore the concordance of the results collected by the same surgeon in the two observation periods; this second phase allows us to estimate the reproducibility of the proposed classification.

### Statistical Analysis

Analyses were performed in R 4.3.2 (R Foundation for Statistical Computing, Vienna, Austria) using the packages irr, irrCAC, DescTools, psych, boot, and pheatmap. Only complete records with valid ordinal scores I–IV were retained. Inter-observer agreement for each session was first quantified using Fleiss’ κ (unweighted) to allow comparability with prior Mason-classification studies [13]. For every κ estimate we computed two-sided 95% confidence intervals by percentile bootstrap (1000 resamples); if bootstrap convergence failed, we used the asymptotic normal approximation based on z = κ/SE(κ). To reflect the ordered nature of the scale and to address potential prevalence/bias effects, we additionally reported Gwet’s AC2 with linear and quadratic ordinal weights, Krippendorff’s α (ordinal weights), the overall pairwise percent agreement (P), and the prevalence- and bias-adjusted κ (PABAK, k = 4). Category-specific Fleiss’ κ (one-versus-all) was also computed for each Mason type. Intra-observer agreement for each rater was assessed by Cohen’s κ (unweighted, linear-weighted, and quadratic-weighted versions) with 95% bootstrap CIs (1000 resamples) and corresponding percent agreement. Agreement levels were interpreted according to Landis & Koch: slight (0.00–0.20), fair (0.21–0.40), moderate (0.41–0.60), substantial (0.61–0.80), almost perfect (0.81–1.00). Values < 0 indicate agreement below chance [14]. To visualise the error structure, we constructed confusion matrices against the majority-vote consensus within each session and displayed row-standardised heat-maps. All tests were two-sided with *p* < 0.05 considered statistically significant. Given 90 subjects and nine raters, standard-error estimates for the principal coefficients were approximately 0.02–0.04, yielding CI half-widths of about 0.05–0.08, consistent with acceptable precision.

## 3. Results

A total of 90 radiographic cases were classified on the Mason–Johnston I–IV scale by nine raters during two independent sessions. Overall inter-observer agreement by unweighted Fleiss’ κ was moderate in both sessions (κ = 0.488, 95% CI 0.42–0.55; and κ = 0.496, 95% CI 0.42–0.56; both *p* < 0.001), with overall pairwise percent agreement 63% and PABAK (k = 4) ≈ 0.50 (Session 1 0.50, 95% CI 0.45–0.56; Session 2 0.51, 95% CI 0.45–0.57; both *p* < 0.001) (Table 1). As weighted, multi-rater ordinal agreement measures, Gwet’s AC2 showed moderate-to-substantial reliability: linear weights 0.618 (95% CI 0.546–0.676) and 0.627 (95% CI 0.557–0.686), and quadratic weights 0.734 (95% CI 0.665–0.787) and 0.745 (95% CI 0.679–0.797) for Sessions 1 and 2, respectively (all *p* < 0.001), consistent with Krippendorff’s α (ordinal) (0.726 and 0.744, Supplementary Table S6). By Mason type, reliability remained moderate for Types II–III (κ ≈ 0.40–0.45) and higher for Type IV (κ ≈ 0.65–0.69), while Type I showed κ ≈ 0.50–0.53 (all *p* < 0.001). Confusion-matrix heat-maps confirmed that most disagreements were between adjacent categories (I/II and II/III), whereas Type IV was rarely misclassified (row-standardised correct classification 0.883 in Session 1 and 0.815 in Session 2; Supplementary Materials).

Intra-observer agreement (Table 2) for individual raters ranged from κ = 0.71 to 0.82 for the unweighted statistic and increased further when using linear or quadratic weights; corresponding percent agreement ranged from 0.80 to 0.88 (all *p* < 0.001 across raters and weightings). These findings confirm moderate to substantial overall reproducibility of the Mason–Johnston classification on plain radiographs, with higher reliability for fracture-dislocation (Type IV) and lower for intermediate Types II–III.

## 4. Discussion

The classification of radial head fractures has evolved over time, with several key systems proposed through the years. Cutler (1926) [15] introduced one of the earliest schemes, dividing fractures into three classes: simple cracks without displacement, fractures with a single separated fragment, and fractures with multiple fragments. A fourth category referred to radial neck fracture [15]. The most influential and enduring scheme was developed by Mason in 1954 [5]. His classification described three types of fracture: Type I, nondisplaced fissures or marginal fractures; Type II, marginal sector fractures with displacement; and Type III, comminuted fractures involving the entire radial head. Mason’s classification aimed to guide treatment, with nonoperative management for Type I, excision for Type III, and variable approaches for Type II. Mason’s classification became the foundation for modern management and remains widely referenced, but subsequent authors sought to address its limitations and improve its clinical applicability.

Johnston (1962) added a fourth type, representing radial head fractures associated with elbow dislocation [6]. This modification was later adopted and popularized by Broberg and Morrey in 1987, who recognized that fracture-dislocations represented a distinct clinical problem [16]. However, including all dislocation-associated fractures under Type IV reduced specificity, as injuries of very different severity were grouped together.

The AO classification system (1990) was designed to provide a comprehensive framework for long bone fractures, including the proximal radius [17]. About proximal radius and ulna, fractures are divided into 3 types: Type A, extra-articular fractures; Type B, intra-articular fractures of one bone; and Type C, intra-articular fractures of both bones. Each type is subdivided into groups and further subgroups reflecting fracture complexity. A key criticism concerns radial head fractures: isolated partial articular fractures may be categorized as simple or multifragmentary, but displaced and nondisplaced fractures fall under the same codes. This limits treatment guidance and outcome evaluation, as clinical management often differs.

Hotchkiss (1997) refined Mason’s scheme focusing on functional impact and emphasizing the presence of mechanical block and surgical feasibility [18]. In his modification, Type I remained nondisplaced or minimally displaced fractures; Type II fractures were displaced (often over 2 mm) and sometimes caused a mechanical block to movement, but were considered amenable reconstruction; Type III fractures were comminuted, and usually required excision or replacement. This classification provided a clearer treatment guidance [19].

Despite moderate reliability, Mason’s classification and its modifications provided by Johnston, remain central in guiding the treatment of radial head fractures.

It is well established that fracture classification is a critical yet challenging tool for standardizing treatment decisions. The most commonly used diagnostic method remains standard radiography due to its low cost, speed, and minimal radiation exposure. However, classification systems based on plain radiographs have demonstrated only inter-observer reliability, especially for radial head fractures [20,21].

In 1997, Morgan et al. evaluated the inter- and intra-observer reliability of the Mason–Johnston classification by analyzing 25 fractures assessed by 20 orthopedic surgeons across two sessions spaced at least three weeks apart [8]. Complete agreement was observed in only 16% of cases [8]. Kappa statistics revealed moderate to poor inter-observer agreement in 69% of cases during the first round and in 45% during the second. Intra-observer agreement was fair to poor in 60% of cases, leading the authors to emphasize the classification’s limited reliability.

Building on this, in 2009, Matsunaga et al. compared three commonly used classification systems, Mason–Johnston, Morrey, and AO/ASIF, by having four observers classify 65 radiographic images on three separate occasions [7]. The Mason–Johnston and Morrey systems demonstrated satisfactory intra-observer agreement, while the AO/ASIF system performed worse. Inter-observer agreement followed a similar pattern, with Mason–Johnston and Morrey showing better consistency than AO/ASIF, thereby reinforcing the relative reliability of the Mason–Johnston classification.

Two years later, Guitton et al. investigated whether 3D CT imaging could improve inter-observer agreement for the Broberg and Morrey classification [10]. Among 85 surgeons evaluating 12 cases, those using 3D CT achieved moderate agreement, whereas the 2D CT group demonstrated poor agreement. However, differences in morphological assessment and treatment recommendations between the two groups were minimal, suggesting that 3D imaging alone may not substantially enhance inter-observer reliability.

Similarly, Sheps et al. found moderate inter-observer agreement for the Hotchkiss classification and poor agreement for the AO/OTA system in a cohort of 43 patients [19]. Agreement improved when certain fracture subtypes were grouped together, indicating better reliability with broader classification categories.

Doornberg et al. also reported excellent intra-observer reliability but only moderate inter-observer agreement for the Broberg and Morrey classification when applied to 119 patients with articular fractures [22].

The addition of an oblique radiographic projection improves inter-observer agreement within the Mason–Johnston classification, as demonstrated by Dillon et al., suggesting that enhanced imaging techniques may contribute to more consistent assessments [23].

Further evaluation by Ayyaswamy et al. revealed poor inter-observer and moderate intra-observer agreement for the Mason–Johnston system [24]. In contrast, the Charalambous classification showed moderate agreement in both categories and allowed for a greater number of fractures to be classified, particularly when morphology rather than displacement was emphasized [2]. However, the observers’ greater familiarity with the Mason–Johnston system and the use of non-standardized radiographs reflecting routine clinical practice were noted as limitations. Despite these factors, the study underscored the limited utility of plain radiographs and highlighted the potential value of more advanced imaging modalities such as CT [20,25]. Supporting this, Rhyou et al. reported high inter- and intra-observer reliability for the Charalambous classification when 3D CT was used, although the study involved only two observers [26].

Finally, the Mason–Johnston classification has been evaluated with respect to its prognostic implications.

Duckworth et al. evaluated 201 patients with radial head and/or neck fractures treated either operatively or nonoperatively [8]. They found that the type of fracture as determined by the Mason–Johnston classification was not associated (*p* = 0.057) with improved Mayo elbow score [8].

The results of our study confirm several critical points previously highlighted in the literature regarding the classification of radial head fractures. Despite the long-standing clinical adoption of the Mason–Johnston classification, its reproducibility remains moderate, particularly in inter-observer assessments. As expected, the highest agreement was observed for Type IV fractures, probably because of their distinct radiographic features, often associated with elbow dislocations, which make diagnosis and classification more straightforward [4,7].

**Figure 1:**
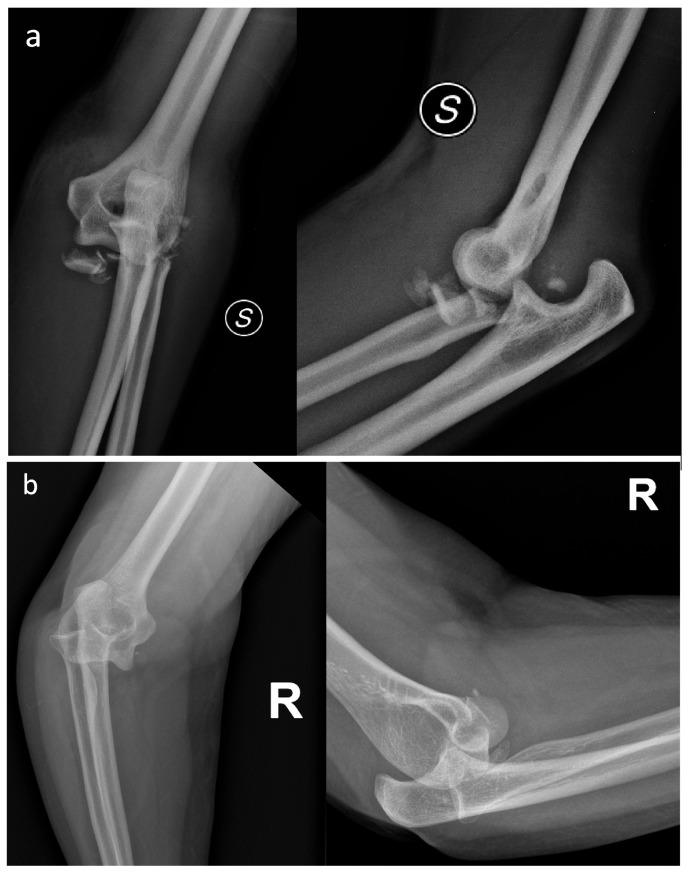
(**a**) Elbow fracture-dislocation associated with a grossly displaced and comminuted radial head fracture. (**b**) Elbow fracture-dislocation associated with a small radial head fracture fragment. Despite both cases involving a radial head fracture associated with elbow dislocation, the treatment and prognosis are different.

Although relatively easy to diagnose using standard radiographs alone, Type IV can present with various patterns of radial head fracture. As a result, the prognostic value and treatment guidance provided by the Mason–Johnston classification for Type IV injuries is quite limited [4,27,28,29] (Figure 1a,b).

Conversely, Types II and III continue to be the most debated categories, with inter-observer agreement ranging from moderate to low [12]. This variability reflects the inherent challenge of reliably assessing displacement, comminution, and joint involvement, parameters that remain subjective when based solely on standard radiographs [20,25].

Intra-observer agreement analysis showed generally good concordance, although variability among observers was noted. This suggests that familiarity with the classification system and individual clinical experience play key roles in consistent fracture assessment. Observers with higher kappa values may have had greater exposure to elbow trauma or more experience in interpreting these fractures, highlighting the significant influence of the human factor on classification reproducibility.

The reliability shown by the Krippendorff alpha variant supports the intra-observer findings, yet it does not entirely address concerns about the Mason–Johnston classification’s role in guiding treatment decisions in borderline cases. While useful for broad clinical communication, its diagnostic limitations may impact therapeutic choices (particularly in Types II and III) where treatment decisions often require nuanced evaluation between conservative and surgical approaches.

### Strengths and Limitations of the Study

One of the primary strengths of this study lies in its robust methodological design. The inclusion of a relatively large sample size (90 cases) and the participation of eight orthopedic surgeons and one radiologist consultant from multiple centers provided a representative and realistic cross-section of clinical practice. This multicentric approach increases the generalizability of the results and allows for a more accurate estimation of both inter- and intra-observer agreement. Additionally, the two-phase design, with a four-month interval between assessments and random reordering of images, minimized recall bias and enhanced the reliability of intra-observer findings. The use of established statistical methods further reinforces the validity of the analysis by offering multiple lenses to evaluate observer concordance.

However, the study also presents several limitations. First, the exclusive reliance on plain radiographs may have constrained the observers’ ability to fully assess fracture morphology, particularly in more subtle or complex cases. Moreover, the observers did not have access to the Greenspan oblique radiographic view, which could have contributed to a more accurate assessment of radial head fractures in selected cases [30]. While this reflects routine clinical conditions, it also underscores the inherent limitations of radiographic classification without the support of advanced imaging. Second, the absence of a training or calibration phase among observers may have amplified inter-observer variability, as individual interpretation of the Mason–Johnston classification criteria may differ. Lastly, this study did not explore the correlation between fracture classification and treatment decisions or clinical outcomes, which limits the conclusions that can be drawn regarding the system’s utility in therapeutic planning.

## 5. Conclusions

This study reinforces the long-standing concerns regarding the reproducibility of the Mason–Johnston classification for radial head fractures. Despite its widespread clinical use, the system demonstrated only moderate inter-observer agreement, particularly in Types II and III fractures, where classification remains most ambiguous. On the other hand, intra-observer agreement was generally higher, although subject to variability, likely reflecting differences in individual interpretation. The highest agreement was observed in Type IV fractures, which tend to have more distinct radiological features and are less susceptible to subjective variation.

These findings suggest that while the Mason–Johnston classification may still serve as a basic communication tool in clinical settings, its diagnostic precision remains limited when based solely on plain radiographs, especially in borderline or complex cases. This study highlights the need for more objective and imaging-supported classification systems that integrate cross-sectional modalities such as CT to improve diagnostic consistency. Future systems should also consider incorporating treatment-oriented frameworks to better support clinical decision-making. Until such systems are developed and validated, caution should be exercised in using the Mason–Johnston classification as a sole guide for therapeutic strategy, particularly in fracture types where morphological interpretation is less straightforward.

## Figures and Tables

**Table 1:** Inter-observer reliability by session and Mason type.

	Kappa	95% CI	*p*-Value
1st OBSERVATION			
Type I	0.50	0.47–0.54	*p* < 0.001
Type II	0.40	0.37–0.44	*p* < 0.001
Type III	0.40	0.36–0.43	*p* < 0.001
Type IV	0.69	0.65–0.72	*p* < 0.001
Overall κ	0.49	0.42–0.55	*p* < 0.001
P (percent agreement)	0.63	-	
PABAK (K = 4)	0.50	0.45–0.56	*p* < 0.001
Krippendorff’s α (ordinal weights)	0.73	0.66–0.79	*p* < 0.001
2nd OBSERVATION			
Type I	0.53	0.49–0.56	*p* < 0.001
Type II	0.41	0.37–0.44	*p* < 0.001
Type III	0.45	0.42–0.49	*p* < 0.001
Type IV	0.65	0.62–0.68	*p* < 0.001
Overall κ	0.50	0.42–0.56	*p* < 0.001
P (percent agreement)	0.63	-	
PABAK (K = 4)	0.50	0.45–0.57	*p* < 0.001
Krippendorff’s α (ordinal weights)	0.74	0.68–0.80	*p* < 0.001

**Table 2:** Intra-observer agreement by rater (unweighted Cohen’s κ and percent agreement).

	Cohen κ (Unweighted)	95% CI (Lower–Upper)	Percent Agreement	*p*-Value
Observer 1	0.82	0.70–0.92	0.88	*p* < 0.001
Observer 2	0.72	0.59–0.83	0.80	*p* < 0.001
Observer 3	0.61	0.48–0.73	0.71	*p* < 0.001
Observer 4	0.73	0.61–0.84	0.81	*p* < 0.001
Observer 5	0.51	0.38–0.65	0.64	*p* < 0.001
Observer 6	0.39	0.26–0.53	0.56	*p* < 0.001
Observer 7	0.62	0.49–0.73	0.72	*p* < 0.001
Observer 8	0.68	0.55–0.79	0.77	*p* < 0.001
Observer 9	0.34	0.20–0.49	0.51	*p* < 0.001

## Data Availability

The data presented in this study are available on request from the corresponding author.

## Supplementary Materials

The following supporting information can be downloaded at https://www.mdpi.com/article/10.3390/jcm14207252/s1. Table S1: Intra-observer agreement by rater—all weights; Table S2: Confusion matrix vs. majority-vote consensus—Session 1 (counts); Table S3: Confusion matrix vs. majority-vote consensus—Session 1 (row proportions); Table S4: Confusion matrix vs. majority-vote consensus—Session 2 (counts); Table S5: Confusion matrix vs. majority-vote consensus—Session 2 (row proportions); Table S6: Inter-observer reliability summary by session (Mason–Johnston I–IV; 90 cases; 9 raters); Figure S1: Misclassification vs. consensus (Session 1); Figure S2: Misclassification vs. consensus (Session 2).
