# Supplementary material for: Radial Head Fractures: Is the Mason Classification Still Effective Today? A Large-Sample Validation of Intra- and Inter-Observer Reliability
Source: J Clin Med. 2025 Oct 14;14(20):7252. doi: 10.3390/jcm14207252 (PMC12565371; doi:10.3390/jcm14207252)
Supplement: Supplementary file 1 [file jcm-14-07252-s001.zip › jcm-3875315-supplementary.pdf]

Supplementary Tables – Reliability Analysis

Supplementary Table S1. Intra-observer agreement by rater—all weights.

Rater	Weight	Cohen κ	95% CI (lower)	95% CI (upper)	p-value
1	unweighted	0.817	0.705	0.916	p < 0.001
1	linear	0.879	0.804	0.938	p < 0.001
1	quadratic	0.932	0.880	0.968	p < 0.001
2	unweighted	0.716	0.592	0.831	p < 0.001
2	linear	0.787	0.669	0.875	p < 0.001
2	quadratic	0.841	0.696	0.930	p < 0.001
3	unweighted	0.610	0.479	0.730	p < 0.001
3	linear	0.698	0.574	0.802	p < 0.001
3	quadratic	0.783	0.676	0.872	p < 0.001
4	unweighted	0.735	0.608	0.843	p < 0.001
4	linear	0.818	0.724	0.906	p < 0.001
4	quadratic	0.890	0.819	0.944	p < 0.001
5	unweighted	0.513	0.380	0.649	p < 0.001
5	linear	0.634	0.523	0.748	p < 0.001
5	quadratic	0.751	0.636	0.836	p < 0.001
6	unweighted	0.392	0.256	0.530	p < 0.001
6	linear	0.506	0.377	0.631	p < 0.001
6	quadratic	0.624	0.482	0.735	p < 0.001
7	unweighted	0.618	0.490	0.733	p < 0.001
7	linear	0.751	0.647	0.835	p < 0.001
7	quadratic	0.849	0.769	0.914	p < 0.001

8	unweighted	0.678	0.554	0.790	$p < 0.001$
8	linear	0.777	0.672	0.860	$p < 0.001$
8	quadratic	0.865	0.788	0.924	$p < 0.001$
9	unweighted	0.337	0.201	0.486	$p < 0.001$
9	linear	0.467	0.339	0.586	$p < 0.001$
9	quadratic	0.602	0.463	0.718	$p < 0.001$

Supplementary Table S2. Confusion matrix vs. majority-vote consensus—Session 1 (counts).

Truth\Rater	1	2	3	4
1	112	33	6	2
2	39	243	44	7
3	0	24	120	18
4	0	6	13	143

Supplementary Table S3. Confusion matrix vs. majority-vote consensus—Session 1 (row proportions).

Truth\Rater	1	2	3	4
1	0.732	0.216	0.039	0.013
2	0.117	0.730	0.132	0.021
3	0.000	0.148	0.741	0.111
4	0.000	0.037	0.080	0.883

Supplementary Table S4. Confusion matrix vs majority-vote consensus—Session 2 (counts).

Truth\Rater	1	2	3	4
1	105	25	3	2
2	46	217	32	2
3	2	37	160	17
4	0	8	22	132

Supplementary Table S5. Confusion matrix vs. majority-vote consensus—Session 2 (row proportions).

Truth\Rater	1	2	3	4
1	0.778	0.185	0.022	0.015
2	0.155	0.731	0.108	0.007
3	0.009	0.171	0.741	0.079
4	0.000	0.049	0.136	0.815

Supplementary Table S6. Inter-observer reliability summary by session (Mason–Johnston I–IV; 90 cases; 9 raters).

Metric (multi-rater)	Session 1 — Estimate (95% CI)	p-value	Session 2 — Estimate (95% CI)	p-value
**Fleiss' κ (unweighted, overall)**	0.488 (0.417–0.551)	$p < 0.001$	0.496 (0.423–0.559)	$p < 0.001$
**Gwet's AC2 (linear weights)**	0.618 (0.546–0.676)	$p < 0.001$	0.627 (0.557–0.686)	$p < 0.001$
**Gwet's AC2 (quadratic weights)**	0.734 (0.665–0.787)	$p < 0.001$	0.745 (0.679–0.797)	$p < 0.001$
**Krippendorff's α (ordinal)**	0.726 (0.654–0.783)	$p < 0.001$	0.744 (0.671–0.795)	$p < 0.001$
**P̄ (pairwise percent agreement)**	0.627	—	0.631	—
**PABAK (k = 4)**	0.503 (0.446–0.564)	$p < 0.001$	0.508 (0.449–0.571)	$p < 0.001$

Supplementary Figure S1. Misclassification vs. consensus (Session 1).

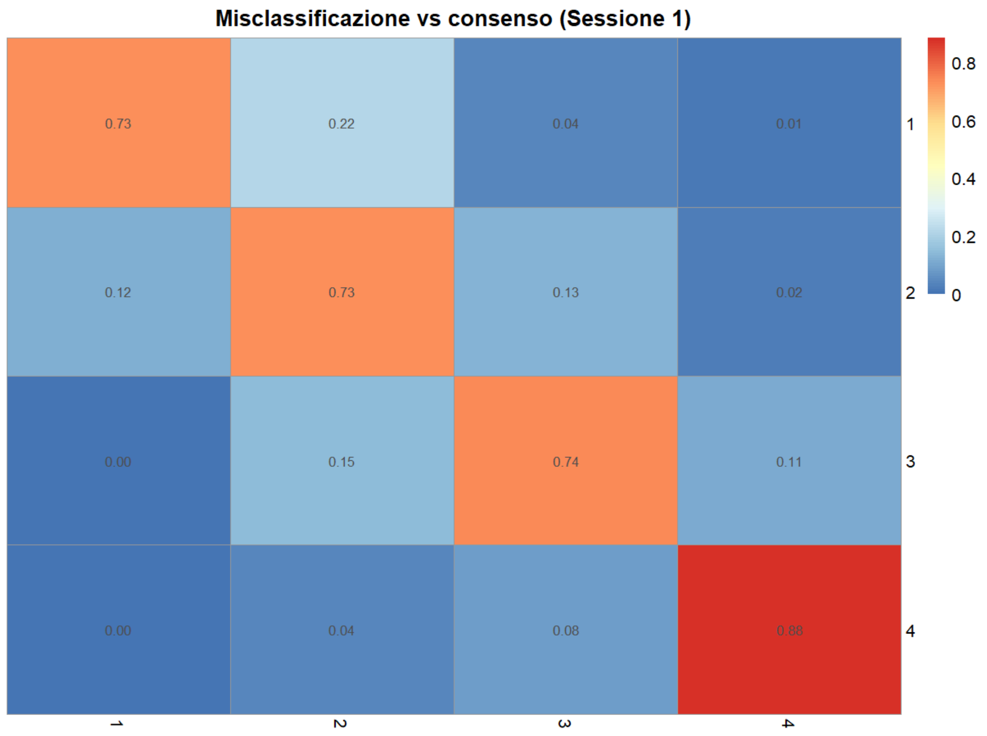

Supplementary Figure S2. Misclassification vs. consensus (Session 2).

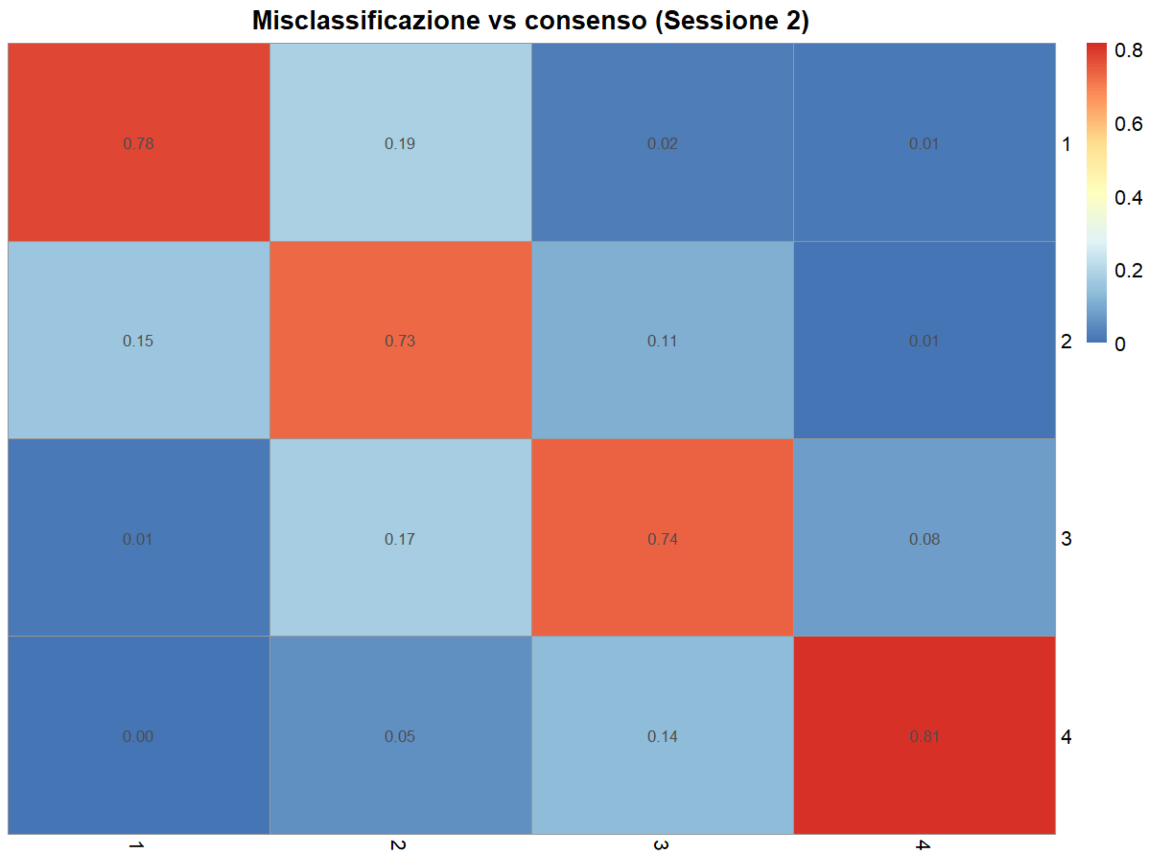

Misclassificazione vs consenso (Sessione 2)
0.78
0.19
0.02
0.01
0.15
0.73
0.11
0.01
0.01
0.17
0.74
0.08
0.00
0.05
0.14
0.81
1
2
3
4
0.8
0.6
0.4
0.2
0
